# Frailty and Social Isolation: Comparing the Relationship between Frailty and Unidimensional and Multifactorial Models of Social Isolation

**DOI:** 10.1177/0898264320923245

**Published:** 2020-06-09

**Authors:** John Maltby, Sarah A. Hunt, Asako Ohinata, Emma Palmer, Simon Conroy

**Affiliations:** 4488 University of Leicester, Leicester, UK

**Keywords:** social isolation, frailty, loneliness, elderly

## Abstract

**Objective:** The aim of the study was to compare uni- and multidimensional models of social isolation to improve the specificity of determining associations between social isolation and frailty. **Methods:** The study included participants aged ≥60 years from the English Longitudinal Study of Ageing assessed for social isolation and frailty (frailty index and Fried phenotype) over a 4-year period. Factor analysis assessed whether social isolation was multidimensional. Multiple regression analysis was used to assess specificity in associations between social isolation and frailty over time. **Results:** Social isolation comprises social isolation from nuclear family, other immediate family, and wider social networks. Over time, social isolation from a wider social network predicted higher frailty index levels, and higher frailty index and Fried phenotype levels predicted greater social isolation from a wider social network. **Discussion:** Social isolation is multidimensional. The reciprocal relationship between social isolation from wider social networks and accumulating frailty deficits, and frailty as a clinical syndrome influencing social isolation from social networks is discussed.

## Introduction

Frailty denotes a decline in function across multiple organ systems, linked to ageing, characterised by vulnerability to poor outcomes in individuals exposed to an apparently innocuous stressor (Fried et al., 2001; Rockwood et al., 1999). There are two main models used to assess frailty: the Fried phenotype, focussing on physical frailty as a pre-disability clinical syndrome (Fried et al., 2001), and a frailty index, assessing the accumulation of deficits (Rockwood et al., 1999). One focus on frailty in the ageing literature has looked at the role of isolation, described as “a calamity of old age” in the medical literature from as far back as 1947 (Rowntree & Nuffield Foundation, 1947; Sheldon, 1947; Tilvis et al., 2011; Yang & Victor, 2011).

Isolation is best understood as two separate, but related, factors: loneliness and social isolation. Loneliness is a subjective, qualitative assessment around a difference between the level of social contact an individual wants and their actual level of contact, when the former exceeds the latter (Veasie et al., 2019). Social isolation is a quantitative assessment reflecting the number of contacts an individual has (Veasie et al., 2019). Loneliness, as an indicator of isolation in the ageing literature, has been linked to a number of health states: heart attacks, strokes, dementia, hospital admissions, and premature deaths (Dreyer et al., 2018; Gale et al., 2018; Hanratty et al., 2018; Kuiper et al., 2015; Shiovitz-Ezra & Ayalon, 2010). Furthermore, research has shown that those adults who are lonely are more likely to be referred to long-term residential or nursing care (Valtorta & Hanratty, 2012). However, social isolation has emerged as being of specific interest in the context of its possible relationship (framed as possibly bidirectional) with frailty, hypothesised as reflecting a syndrome of enforced withdrawal resulting from a cycle of energy dysregulation or lack of interest or capability in social engagement (Bandeen-Roche et al., 2006; Fried et al., 2001; Singleton et al., 2017). The relationship between social isolation and the two models of frailty has demonstrated inconsistent results. Social isolation has been found to predict higher risk of worsening frailty when phenotype is used to measure frailty (Makizako et al., 2018), albeit sometimes just among men (Gale et al., 2018), but has not been found to predict a change in the frailty index over time (Gale et al., 2018). Furthermore, the frailty phenotype has been found to predict high levels of social isolation after 2 years, and the frailty index has predicted an increased risk of social isolation after 4 years (Gale et al., 2018).

There have been a few studies that have used data from the English Longitudinal Study of Ageing (a study that collects multidisciplinary data from a sample of people aged 50 and older [Banks et al., 2019]) to explore the effects of social isolation on health outcomes. Researchers measuring social isolation using data from this study have found it to be related to a number of outcomes in addition to frailty (e.g. mortality, smoking, blood pressure, and fibrinogen levels) (Shankar et al., 2011; Steptoe et al., 2013) but have treated it as a single dimension. However, theory and research suggest that social isolation is multifactorial (Cornwell & Waite, 2009; Lubben, 1988; Lubben et al., 2006). For example, based on indicators assessing levels of social connectedness, participation, and support among older adults, from the National Social Life, Health, and Ageing Project, it is suggested that social isolation reflects two dimensions. The first is the size of the individual’s network size (i.e. number of contacts). The second is the amount of activity the individual engages in within that network (i.e. amount of contact) (Cornwell & Waite, 2009). Therefore, given that previous studies using the English Longitudinal Study of Ageing explored the relationship between social isolation and frailty using just a single dimension of social isolation, there is an opportunity to explore the nature of that relationship using a multidimensional model of social isolation. For example, when finding that unidimensional assessments of social isolation predict frailty (Makizako et al., 2018), further specificity could be afforded through the consideration of whether there are multiple dimensions (e.g. contact with different people; family or friends). The identification of dimensions which best predict frailty could better inform social support interventions by targeting particular associations. Furthermore, where unidimensional assessments of social isolation are not found to predict frailty or to partially predict frailty in some groups and not others (Gale et al., 2018), it may be that the effects of social isolation on frailty are being masked or weakened by the combination of dimensions of social isolation that do not predict frailty with dimensions that do.

Evidence from large-cohort datasets based on unidimensional models suggests that social isolation and frailty can be related over time. However, current evidence suggests that social isolation is multidimensional. It is proposed that the operationalisation of multidimensional models of social isolation may increase the specificity around the relationship between social isolation and frailty in large-cohort datasets. Such specificity would be important for informing policy and interventions targeting social isolation and frailty and considering an individual’s social placement in terms of their social isolation, thereby lessening some of the disadvantages that emerge from this relationship between social isolation and frailty. The aim of the current study was twofold: to examine whether (1) a multidimensional structure existed among the social isolation indicators in a large-cohort dataset and (2) a multidimensional, rather than a unidimensional, model of social isolation would improve the specificity of the relationship between social isolation and frailty longitudinally.

## Method

### Participants

Data were obtained from Wave 1 (original *n* = 12,099), Wave 2 (original *n* = 9432), and Wave 4 (original *n* = 11,050) of the English Longitudinal Study of Ageing dataset (Banks et al., 2019). The original dataset comprises data on a representative sample of people 50 and older, living in private households in England (Banks et al., 2019). We created datasets for individuals 60 years or older at Wave 2 to examine the longitudinal effects (from Waves 2 to 4) of social isolation on frailty as this created a gap of 4 years between the waves, the same as or similar to the time frames reported by a recent research into the longitudinal effects of social isolation on frailty (Gale et al., 2018; Makizako et al., 2018).

## Measures

### Social Isolation

To assess social isolation, we used five indicators used by the previous research (Gale et al., 2018; Shankar et al., 2011; Steptoe et al., 2013): (1) being unmarried or not cohabiting, (2) having less than monthly contact with their children, (3) having less than monthly contact with other family members, (4) having less than monthly contact with friends (whether face-to-face, written, or by telephone), and (5) not being a member of any social organisations (e.g. social groups).

Previous studies (Gale et al., 2018; Shankar et al., 2011; Steptoe et al., 2013) created a social isolation index by allocating one point for “being unmarried or not cohabiting, having less than monthly contact (whether face-to-face, written, or telephone) with each of children, other members of the family, and friends, and not being a member of organizations such as religious groups, evening classes, social groups, or residents associations”. Possible scores ranged from 0 to 5, with higher values indicating higher levels of social isolation. However, we introduced a different way of scoring 4 of these 5 items because the scoring system presented by previous authors (Gale et al., 2018; Shankar et al., 2011; Steptoe et al., 2013) did not seem precise in terms of assessing the amount of social isolation experienced by individuals. For the first indicator (“being unmarried or not cohabiting”), it is not necessarily the case that unmarried or non-cohabiting individuals are socially isolated, as they may be living with their children, another family member, or a friend. Therefore, we changed the scoring of this variable to include individuals who were (a) unmarried or not cohabiting and (b) did not have children, another family member, or a friend living with them at home. Therefore, this became an indicator of “living alone”, which has previously been recognised as an indicator of social isolation (Makizako et al., 2018), and for which respondents were given a score of “1”.

The other three changes were based on three items normally scored based on “having less than monthly contact (whether face-to-face, written, or by telephone) with each of their children [item 2], other members of the family [item 3], and friends [item 4]” (e.g. Gale et al. (2018)). In the English Longitudinal Study of Ageing dataset, respondents are actually asked about contact with people “not counting anyone who lives with you”. Therefore, individual use of these items may indicate individuals who have less than monthly contact with their children, family, and friends, but potentially have a child, family member, or friend at home (in addition, individuals may cite this survey question as not applicable because they have no children, family, or friends living away from them but do have a child, family member, or friend who is a resident in their home). Finally, individuals without children, family members, or friends would not answer this question. Therefore, we changed the criteria for giving a score of “1” on this item to include individuals who did not have

1. Children living at home with them AND had less than monthly contact (whether face-to-face, written, or by telephone) with any and all children not living with them [item 2],
2. Family members living at home with them AND had less than monthly contact (whether face-to-face, written, or by telephone) with any/all family members not living with them [item 3], and
3. Friends living at home with them AND had less than monthly contact (whether face-to-face, written, or by telephone) with any and all friends not living with them [item 4].

Therefore, we produced two sets of variables. The first set was based on the inclusion of the variables used to produce an overall score for the five indicators (named our unidimensional model) suggested by the previous research (Gale et al., 2018; Shankar et al., 2011; Steptoe et al., 2013). The second set was based on our different scoring system, used to explore the multidimensional nature of these five items.

### Frailty

We calculated two frailty assessments, reflecting the two main models of frailty: the Fried phenotype of physical frailty and a frailty index. We also calculated a five-item index of the Fried phenotype of physical frailty as indicated by the presence of (scored “1” if present and “0” if not present) five indices of frailty (Fried et al., 2001; Gale et al., 2018; Hughes et al., 2004). This five-item index comprised (1) loss of ≥10% body weight or low body mass index (BMI; <18.5 kg/m^2^), (2) falling in the lowest 20% of the distribution of grip strength (taking gender and BMI into account), (3) falling into the slowest 20% of the distribution of gait speed (after taking gender and height into account), (4) falling in the lowest 20% of the distribution of the amount of exercise in terms of the frequency (“vigorous”, “moderate”, or “mild exercise”), and (5) exhaustion, based on a positive response to either of two questions on the Center for Epidemiologic Studies Depression Scale (CES-D) (Radloff, 1977), namely “Felt that everything I did was an effort in the last week” or “Could not get going in the last week”. The Fried phenotype assigns possible scores of 0–5 and groups subjects into three categories that indicate increasing frailty scores, namely “non-frail” if scoring 0, “pre-frail” if scoring 1–2, and “frail” if scoring 3–5. We computed a frailty index using all 52 indices suggested by Gale et al. (2018) that reflect problems with everyday activities (e.g. dressing), illness or disease (e.g. stroke), self-ratings for different aspects of health (e.g. self-rated eyesight), and cognitive function (e.g. naming the correct day). The frailty index score is calculated by summing the number of deficits present for each participant and then dividing by the number of deficits considered, giving a score that ranges between 0 and 1.

### Covariates and Confounds

We included several variables to account for covariate and confounding effects. These variables included gender, age, whether the individual had ever smoked, and educational attainment (Wave 1), and current wealth (value of current accounts, savings and investments, value of own property, or business assets, net debt, and excluding pension assets) (Wave 2). We also included two indices of mental well-being at Wave 2. We calculated a depression score using an amended version of the eight-item CES-D (Radloff, 1977). The amendment involves omitting three items from the calculation of an overall depression score, two of which are used as indicators of exhaustion when deriving the physical frailty phenotype and one of which refers to loneliness (Gale et al., 2018). Furthermore, Gale et al. had noted dissatisfaction with controlling just for depression using this recalculated version of CES-D, as it could have some overlap with the social isolation and frailty constructs, with each of the measures having similar items. Therefore, based on the findings that social isolation is associated with a number of mood disorders and psychiatric conditions (Chou et al., 2011; Han et al., 2018; Li et al., 2017), we created an additional variable, indicating whether the individual had reported any of a set of psychiatric conditions (hallucinations, anxiety, depression, emotional problems, schizophrenia, psychosis, mood swings, and manic depression) in the previous two years, at Wave 2. We also obtained scores for the four-item revised UCLA loneliness scale (Hughes et al., 2004) at Wave 2. This scale assesses the frequency (1 = “hardly ever or never” to 3 = “often”) with which the participant lacks companionship, feels isolated, or feels left out (Wave 2).

### Statistical Analysis

The statistical analysis strategy followed the two objectives of the study.

To examine the first objective, of whether a multidimensional structure existed among the social isolation indicators, we examined the underlying structure of the social isolation indicators among 4918 cohort members (Sample 1) who provided a full data profile for the five indicators of social isolation at Wave 2. We used principal components analysis to identify one or more variables from a larger set of variables. Principal component analysis provides an evaluation of structural validity, the degree to which the scoring of an assessment adequately reflects the dimensionality of the construct (Messick, 1995). Principal components analysis comprises two stages: (1) extraction: summarising the total variance shared between the five social isolation variables within a smaller set of variance components to determine how many latent dimensions occur; and (2) rotation: identifying the dimension on which each of the social isolation indicators is positioned. The aim of this analysis was to achieve a “simple structure” in which each indicator is clearly allocated to a particular dimension (Thurstone, 1947). This analysis tested whether the five social isolation indicators were unidimensional or multidimensional. To assess the number of dimensions to the five social isolation variables, we applied a principal component analysis, using polychoric correlations, as these correlations should be used when item responses are dichotomous. We then used parallel analysis. This analysis compares two sets of eigenvalues (indices of variance accounted for by possible underlying dimensions), the first calculated from the dataset and the second from a Monte Carlo simulation which calculates eigenvalues generated from random data. To determine how the social isolation indicators were positioned on each dimension, we used promax rotation. The position of each indicator on each dimension can be assessed against the criteria of .55 ≤ *x* < .63 (“good”), .63 ≤ *x* < .71 (“very good”), and ≥.71 (“excellent”) (Tabachnick & Fidell, 2007). The reported findings demonstrate a simple structure, with each item loading above “excellent” on one component and “not relevant” on the remaining components.

Secondly, to examine the second objective, whether a multidimensional model of social isolation would increase the specificity of the relationship between social isolation and frailty longitudinally, we ran a series of multiple regression analyses. These formed two sets of analyses, each with two subsamples. The first set of analyses was used to examine which social isolation dimensions predicted frailty longitudinally, for which we were able to create two subsamples in two separate datasets. The first subsample comprised 2192 participants (of the 4918 cohort members) (Sample 2) for whom we were able to obtain a full data profile for the 52 indices used to assess the frailty index (Waves 2 and 4) and the demographic, covariate, and confounding variables (Waves 1, 2, and 4). The second subsample contained 1662 participants (Sample 3) for whom we were able to obtain a full data profile for the five indices used to assess the frailty phenotype (Waves 2 and 4) and the demographic and covariate variables (Waves 1, 2, and 4). These multiple regression models examined which dimensions of social isolation predicted scores on either the frailty index (*n* = 2192) or the Fried frailty phenotype (*n* = 1662) at Wave 4, after controlling for the respective scores for frailty at Wave 2 and for the demographic and covariates/confounds. The second set of multivariate regression analyses was used to examine how frailty dimensions (either frailty index or phenotype) predicted aspects of social isolation (overall score, or social isolation from nuclear family, other immediate family, or wider social network) over time. This analysis was run on the sample for whom we were able to obtain a full data profile for the social isolation measure (Waves 2 and 4), frailty (index or phenotype at Wave 2), and the demographic, covariate, and confounding variables (Waves 1, 2, and 4). Because of further missing data relating to social isolation at Wave 4, we created two further subsamples (frailty index, Sample 4, *n* = 1457; Fried phenotype, Sample 5, *n* = 1131) from the two separate datasets used for the first set of analyses. These models examined whether the method of frailty measurement (index or phenotype) predicted scores on either the unidimensional or multidimensional aspects of social isolation at Wave 4, after controlling for the respective scores for social isolation at Wave 2 and for demographic and covariate variables. To ensure that all the variables entered at either Step 1 or 2 of the regression series were independent of each other (so as not to inflate the variance of at least one of the regression coefficients due to collinearity), variance inflation and tolerance factor statistics were calculated when possible.

## Results

Table 1 provides descriptive and frequency statistics relating to the social isolation, frailty, demographic, and covariate variables for all subsamples reported in this article.

**Table 1.** Mean (*SD*) or Frequency Statistics for Frailty, Loneliness, Demographic, and Confounds/Covariates for Waves 2 and 4 for the Five Study Samples Reported.

	Sample 1 (*n* = 4918)	Sample 2 (*n* = 2192)	Sample 3 (*n* = 1662)	Sample 4 (*n* = 1457)	Sample 5 (*n* = 1131)
Gender	2231 males (45.36%) and 2687 females (54.64%)	1008 males (45.99%) and 1184 females (54.01%)	775 males (46.63%) and 887 females (53.37%)	773 males (53.05%) and 684 females (46.95%)	606 males (53.58%) and 525 females (46.42%)
Age (years)	70.52 (7.55)	69.12 (6.75)	68.96 (6.60)	69.12 (6.75)	67.75 (5.97)
Highest educational qualification^a^		(i) *n* = 271 (12.36%), (ii) *n* = 284 (12.96%), (iii) *n* = 120 (5.47%), (iv) *n* = 383 (17.47%), (v) *n* = 124 (5.66%), (vi) *n* = 216 (9.85%), and (vii) *n* = 794 (36.22%)	(i) *n* = 205 (12.33%), (ii) *n* = 224 (13.47%), (iii) *n* = 92 (5.54%), (iv) *n* = 309 (18.59%), (v) *n* = 87 (5.23%), (vi) *n* = 159 (5.23%), and (vii) *n* = 586 (35.26%)	(i) *n* = 208 (14.28%), (ii) *n* = 201 (13.80%), (iii) *n* = 76 (5.22%), (iv) *n* = 255 (17.50%), (v) *n* = 100 (6.86%), (vi) *n* = 146 (10.02%), and (vii) *n* = 471(32.33%)	(i) *n* = 158 (13.97%), (ii) *n* = 160 (14.15%), (iii) *n* = 61 (5.39%), (iv) *n* = 213 (18.83%), (v) *n* = 68 (6.01%), (vi) *n* = 114 (10.08%), and (vii) *n* = 357 (31.56%)
Net wealth (£)		229038.72 (279355.55)	240835.71 (299416.72)	264138.19 (315714.06)	271327.12 (324166.94)
Whether had ever smoked^a^		*n* = 1358 (61.95%)	*n* = 1012 (60.89%)	*n* = 915 (62.80%)	*n* = 688 (60.83%)
Psychiatric problems in the last two years		*n* = 52 (2.37%)	*n* = 32 (1.93%)	*n* = 29 (1.99%)	*n* = 14 (1.24%)
Depressive symptoms		.84 (1.16)	.79 (1.11)	.71 (1.01)	.66 (.94)
Loneliness		5.62 (1.64)	5.53 (1.59)	5.27 (1.41)	5.22 (1.36)
Frailty (Wave 2)		.12 (.10)^b^	.63 (.63); non-frail, *n* = 758 (45.61%); pre-frail, *n* = 768 (46.21%); frail, *n* = 136 (8.18%)^c^	.11 (.09)^b^	.55 (.60); non-frail, *n* = 568 (50.22%); pre-frail, *n* = 500 (44.21%); frail, *n* = 63 (5.57%)
Frailty (Wave 4)		.11 (.09)^b^	.61 (.65); non-frail, *n* = 799 (48.07%); pre-frail, *n* = 706 (42.48%); frail, *n* = 157 (9.45%)^c^	.09 (.08)^b^	.54 (.61); non-frail, *n* = 589 (52.08%); pre-frail, *n* = 470 (41.56%); frail, *n* = 72 (6.37%)^c^

a Wave 1.

b Frailty index.

c Fried phenotype: (i) degree or equivalent; (ii) higher education, but below degree; (iii) a level or equivalent; (iv) GCE O level equivalent; (v) CSE or equivalent; (vi) other; (vii) no qualification.

### Principal Component Analysis (Sample 1)

For the principal component analysis, the parallel analysis revealed, at extraction, the fourth highest eigenvalue (1.483, 1.338, 1.004, and .606) failed to exceed the fourth mean eigenvalue (1.040. 1.018, .999[recurring], and .982), suggesting three dimensions to the social isolation indicators. These findings relating to the rotated solution are presented in Table 2. Component 1 characterises social isolation from a nuclear family, through being unmarried or non-cohabiting, living alone, and being socially isolated from any children. Component 2 describes social isolation from other immediate family, that is being socially isolated from parents and siblings. Component 3 characterises social isolation from a wider social network, that is being socially isolated from friends and social organisations. Correlations between the three components did not exceed *r* = .09. A shared variance of no more than .8% suggests the components are independent of each other. Therefore, we introduced three social isolation subscales, adding items when multiple items loaded on the same component to form (1) “social isolation from a nuclear family” (from the living alone and social isolation from one’s children variables), (2) “social isolation from other immediate family” (from the social isolation from wider family variables), and (3) “social isolation from a wider social network” (from the social isolation from friends and social organisation variables). The mean scores, standard deviations (*SD*s), and ranges for each of these scales are also presented in Table 2.

**Table 2.** Principal Component Analysis with Promax Rotation of the Five Social Isolation Variables. Mean, *SD*, and Range of Scores for the Social Isolation Scales (Sample 1, *n* = 4918).

	Three Factors		
	1	2	3
Unmarried or non-cohabiting/living alone	**.88**	.01	−.18
Social isolation from one’s children	**.79**	−.01	.23
Social isolation from wider family	.01	.01	**.97**
Social isolation from friends	.05	**.83**	−.14
Social isolation from social organisations	−.04	**.84**	.15

	Minimum score	Maximum score	Mean (*SD*)
Social isolation from nuclear family	0	2	.39 (.61)
Social isolation from other immediate family	0	1	.15 (.36)
Social isolation from a wider social network	0	2	.28 (.51)

*Note. SD* = standard deviation. Bolded numbers represent important loadings.

### Social Isolation Predicting Frailty (Samples 2 and 3)

Across the multiple regression analyses, we used the two definitions of social isolation from the same five aforementioned social isolation variables: (1) the three-factor multidimensional definition based on the principal component analysis findings (social isolation from nuclear family, other immediate family, and wider social network), and, as comparison, (2) an overall score representing a unidimensional definition based on previous formulations of social isolation (Gale et al., 2018; Shankar et al., 2011; Steptoe et al., 2013). Across the regression analysis, for all the samples (samples 2 and 3, and those to be reported in the next section, namely samples 4 and 5), the variance inflation and tolerance factor statistics were no larger than 1.25 and no smaller than .8 respectively, falling within the criteria of variance inflation factors being less than 5 and tolerance factors more than .2 (Kutner et al., 2004). Therefore, these variables could be assumed to be independent.

The frailty index scores (Sample 2) were positively skewed (Wave 2, skewness = 1.55; Wave 4, skewness = 1.44), falling outside the criteria of ±1 representing “very good” symmetry, and were therefore log-transformed for the regression analysis, with .01 added to avoid logarithms of zero. Based on these criteria, the Fried phenotype scores (Sample 3) were not skewed (Wave 2, skewness = .50; Wave 4, skewness = .69, kurtosis = −.65) and therefore were not log-transformed for the regression analysis.^1^ The multidimensional measures of social isolation at Wave 2 were all positively skewed (frailty index [Sample 2], nuclear family skewness = 1.37, other immediate family skewness = 2.12, and social network skewness = 1.66; Fried phenotype [Sample 3], nuclear family skewness = 1.39, other immediate family skewness = 2.14, and social network skewness = 1.73), but the unidimensional measure was not (frailty index [Sample 2], skewness = .90; Fried phenotype [Sample 3], skewness = .89). Therefore, the multidimensional measures of social isolation were also log-transformed for the regression analysis, with .01 again added to avoid logarithms of zero.

For the frailty index (Sample 2), both the unidimensional and multidimensional models of social isolation in Step 1 (due to the variables entered in this step being the same) were significant (*F*[9, 2182] = 345.34, *r* = .77; *r*^2^ = .59, adj *r*^2^ = .59, *p* < .001), with being older, having higher levels of frailty at Wave 2, or having higher levels of loneliness all demonstrating statistical significance in predicting frailty index scores at Wave 4. The introduction of the multidimensional social isolation measures created a significant change in how the multidimensional model of social isolation predicted the frailty index scores (Δ*F* = 2.65, *p* = .047), with social isolation from a wider social network predicting unique variance in the frailty index at Wave 4 (Table 3). However, the unidimensional social isolation measure failed to produce a significant change in any of the regressions and, therefore, did not predict frailty at Wave 4 (unidimensional score: frailty index, Δ*F* = 2.25, *p* = .134).

**Table 3.** Regression Analysis with Frailty at Wave 4 Used as the Dependent Variable, and Social Isolation, Frailty, and Covariate/Confounds (Wave 2) Used as Predictor Variables.

	B	β	*t*	*p*	Lower Bound CI (95%)	Higher Bound CI (95%)
Step 1						
Age	.02	.12	8.67	.000	.011	.018
Gender	.04	.02	1.60	.111	−.008	.082
Frailty (Wave 2)	.78	.69	44.39	.000	.750	.819
Educational qualification	−.01	−.03	−1.93	.054	−.021	.000
Smoking	.03	.02	1.13	.260	−.019	.071
Depressive symptoms	.01	.02	1.27	.205	−.007	.034
Psychiatric symptoms	−.09	−.02	−1.25	.210	−.234	.051
Wealth	−.01	−.03	−1.86	.063	.000	.000
Loneliness	.02	.04	2.45	.014	.004	.033
Step 2						
SI from a nuclear family	.02	.01	.85	.395	−.021	.054
SI from other immediate family	−.05	−.02	−1.40	.161	−.107	.018
SI from a wider social network	.06	.04	2.53	.012	.014	.107

*Abbreviations:* SI = social isolation; B = unstandardised beta; β = standardised betas; *t* = *t* test value; *p* = probability; CI = confidence interval.

*Note*. Frailty at Wave 4 is used as a dependent variable, and gender, age, frailty at Wave 2, education level, whether smoker during lifetime, depressive symptoms, psychiatric symptoms in the last two years, wealth, and loneliness (Step 1), and social isolation (Step 2) are used as predictor variables (Sample 2, *n* = 2192).

For the Fried phenotype (Sample 3), both the unidimensional and multidimensional models of social isolation in Step 1 (due to the variables entered in this step being the same) were significant (*F*[9, 1652] = 66.48, *r* = .52; *r*^2^ = .27, adj *r*^2^ = .26, *p* < .001), with a higher age, frailty at Wave 2, lower educational attainment, depression, lower wealth, and loneliness predicting the Fried phenotype of frailty at Wave 4. However, neither the introduction of the unidimensional nor that of the multidimensional social isolation measure created a significant change in the respective model (multidimensional, Δ*F* = 1.11, *p* = .344; unidimensional, Δ*F* = .52, Δ*R* = .001, *p* = .473), suggesting that neither of the formulations of social isolation predicted frailty.

### Frailty Predicting Social Isolation (Samples 4 and 5)

This analysis comprised two sets of regressions. For the first set, we ran two-step multiple regressions for social isolation from one’s nuclear family and a wider social network, with a unidimensional social dimension as the outcome variable. For the second set of tests, we ran two-step logistical regressions for social isolation from other immediate family as the outcome variable (due to the scoring of this variable consisting of two categories). Within this analysis, we examined whether either the frailty index or the Fried phenotype (Step 2) predicted scores on the social isolation measures (social isolation from (1) a nuclear family, (2) other immediate family, (3) wider social network, and (4) unidimensional social isolation at Wave 4, after controlling for the respective measure of social isolation, and demographic and covariate variables, at Wave 1 or 2 (Step 1). As with the first set of analyses, due to a positive skew, the multidimensional measures of social isolation and the frailty index were log-transformed for the regression analysis, with .01 added to avoid logarithms of zero.

Our main analysis focussed on the link between social isolation from a wider social network and frailty. Therefore, we present the findings on this firstly. For social isolation from a wider social network, for both assessments of frailty, in Step 1, the models were significant (frailty index dataset, *F*[9, 1447] = 65.91, *r* = .54; *r*^2^ = .29, adj *r*^2^ = .29, *p* < .001; Fried phenotype dataset, *F*[9, 1121] = 61.25, *r* = .57; *r*^2^ = .33, adj *r*^2^ = .32, *p* < .001). In both samples (samples 4 and 5), at Step 1, higher levels of social isolation from a wider social network at Wave 2, higher levels of education (Wave 1), and lower wealth at Wave 2 predicted higher levels of social isolation from a wider social network at Wave 4. In addition, higher levels of loneliness predicted higher levels of social isolation among the frailty index subsample (Sample 4), and being male predicted higher levels of social isolation from a wider social network at Wave 4 among the Fried phenotype subsample (Sample 5). At Step 2, both the frailty index (Δ*F* = 6.28, *p* = .012) and the Fried phenotype (Δ*F* = 5.18, *p* = .023) at Wave 2 predicted social isolation from a wider social network at Wave 4 (Table 4).

**Table 4.** Regression Analysis with Social Isolation as the Dependent Variable (Wave 4), and Frailty, Social Isolation, and Covariate/Confounds (Wave 2) Used as Predictor Variables.

	B	β	*T*	*p*	Lower Bound CI (95%)	Higher Bound CI (95%)	B	β	*t*	*p*=	Lower Bound CI (95%)	Higher Bound CI (95%)
	Frailty index (*n* = 1457)						Frailty phenotype (*n* = 1131)					
	Sample 4						Sample 5					
Step 1												
Age	.01	.02	.68	.494	−.010	.020	.01	.03	1.10	.274	−.007	.026
Gender	−.18	−.04	−1.84	.067	−.362	.012	−.23	−.06	−2.18	.029	−.440	-.023
Social isolation from a wider social network (Wave 2)	.50	.48	21.02	.000	.449	.541	.54	.52	20.76	.000	.490	.592
Educational qualification	−.11	−.12	−5.11	.000	−.155	−.069	−.10	−.11	−4.14	.000	−.147	-.052
Smoking	.01	.01	.05	.961	−.182	.191	.10	.02	.96	.339	−.105	.306
Depressive symptoms	.01	.01	.03	.975	−.094	.097	.02	.01	.34	.736	−.092	.130
Psychiatric symptoms	.05	.01	.15	.883	−.591	.687	.24	.01	.52	.601	−.653	1.129
Wealth	−.01	−.05	−2.11	.035	.000	.000	−.01	−.06	−2.40	.016	.000	.000
Loneliness	.07	.05	2.04	.041	.003	.136	.03	.02	.69	.493	−.049	.102
Step 2												
Frailty (Wave 2)	.18	.06	2.51	.012	.040	.328	.20	.06	2.28	.023	.028	.372

*Abbreviations*: B = unstandardised beta; β = standardised betas; *t* = *t* test value; *p* = probability; CI = confidence interval.

*Note*. Social isolation from a wider social network at Wave 4 is used as a dependent variable, and gender, age, social isolation from a wider social network at Wave 2, education level, whether smoker during lifetime, depressive symptoms, psychiatric symptoms in the last two years, wealth, and loneliness (Step 1), and frailty at Wave 2 (frailty index/Fried phenotype) (Step 2) are used as predictor variables.

For all the other outcome variables (social isolation from nuclear family, social isolation from other immediate family, and unidimensional social isolation), though we found that the regression models were significant at Step 1, neither of the measures of frailty predicted any of these aspects of social isolation at Step 2. A summary of these statistics is provided in Table 5.

**Table 5.** Summary Findings from Regression Analysis Models that were not Statistically Significant with Social Isolation Variables.

	Frailty Index (*n* = 1457)			Fried Phenotype (*n* = 1131)		
	Sample 4			Sample 5		
	Step 1	Unique Variance Predictors at Step 1	Step 2	Step 1	Unique Variance Predictors at Step 1	Step 2
1. Social isolation from nuclear family (Wave 4)^∗^	*F*[9, 1447] = 178.60, *r* = .73; *r*^2^ = .53, adj *r*^2^ = .52, *p* < .001	Male and social isolation from a nuclear family at Wave 2	Δ*F* = .89, *p* = .346	*F*[9, 1121] = 147.72, *r* = .74; *r*^2^ = .54, adj *r*^2^ = .54, *p* < .001	Male and social isolation from a nuclear family at Wave 2	Δ*F* = .26, *p* = .612
2. Social isolation from extended family (Wave 4)^**^	χ^2^ = 140.47, Nagelkerke *r*^2^ = .17, *p* < .001	Social isolation from extended family at Wave 2	Δχ^2^ = .21, *p* = .886	χ^2^ = 113.82, Nagelkerke *r*^2^ = .17, *p* < .001	Male and social isolation from extended family at Wave 2	Δχ^2^ = .98, *p* = .323
3. Unidimensional social isolation (Wave 4)^∗^	*F*[9, 1447] = 53.66, *r* = .50; *r*^2^ = .25, adj *r*^2^ = .25, *p* < .001	Higher age, higher social isolation at Wave 2, and lower educational qualifications	Δ*F* = .69, *p* = .408	*F*[9121] = 49.93, *r* = .54; *r*^2^ = .29, adj *r*^2^ = .28, *p* < .001	Higher social isolation at Wave 2, lower educational qualifications, and lower wealth	Δ*F* = 3.77, *p* = .052

*Abbreviations*: * = multiple regression analysis; ** = logistic regression analysis.

*Note*. Unidimensional, nuclear family, and extended family social isolation at wave four is used as dependent variable, and social isolation, demographic, and covariates at Waves 1 and 2 (Step 1), and either frailty index or Fried phenotype (Step 2) are used as predictor variables.

1 Though some authors (e.g. Gale et al., 2018) treat the Fried phenotype variable as a nominal variable (a variable with no order to the scoring), the Fried phenotype categories are directly based on the ordering of continuous variables, and also the categories assigned to levels of frailty are also in an order. Therefore, in this analysis, we treat this variable as an ordered variable in the analysis. This also allows us to compare the separate model contributions within and across the different regression models and examine the additional variance accounted for by the social isolation and frailty variables, after controlling for possible confounding variables.

## Discussion

The first finding from our analysis suggests that social isolation, as assessed within the English Longitudinal Study of Ageing and by items suggested by Gale et al. (2018), is multidimensional, reflecting three dimensions, namely social isolation from, “nuclear family”, “other immediate family”, and “a wider social network”. The second finding is that, of these three social dimensions, social isolation from a wider social network predicts one aspect of frailty (the frailty index) over a four-year period, when controlling for frailty at the baseline as well as several other possible confounders (and possible interactions therein). Furthermore, both the frailty index and the Fried phenotype predicted social isolation from a wider social network over the same period, when controlling for social isolation and other confounds at baseline.

The finding that social isolation is multifactorial is not new. However, the current finding suggests a divergence from previous practice of using these five variables within the English Longitudinal Study of Ageing as a single dimension (Gale et al., 2018; Shankar et al., 2011; Steptoe et al., 2013), and the use of principal components analysis to determine this presents structural validity for this multidimensional model. The current formulation of the latent components to these five variables focusses more on a biological and social kinship structure based around social isolation from the nuclear family, other immediate family, and wider friendships and social organisations. Therefore, this finding suggests that the treatment of social isolation as a unidimensional concept in previous studies using the English Longitudinal Study of Ageing data may have masked some of the effects relating to social isolation, and specifically social isolation from a social network. This formulation of social isolation as multidimensional in the English Longitudinal Study of Ageing has allowed greater specificity than in other studies that have found a relationship between social isolation and frailty over at least a 4-year period (Gale et al., 2018). This has led to our findings that suggest it is social isolation from a wider social network that is related to frailty over time.

The results suggest that social isolation from a wider social network is integrated with the accumulation of deficits (as indicated by the frailty index) and that this interaction emerges over time from a cycle of energy dysregulation or social embarrassment, or a lack of interest or capability in social engagement (Bandeen-Roche et al., 2006; Singleton et al., 2017), aspects that are not assessed here. The findings also suggest that frailty as a pre-disability clinical syndrome predicting social isolation is the emergence of this phenotype, potentially indicating a critical threshold for the physiologic reserve and the beginning of a social isolation process from wider social networks. Therefore, measuring both multidimensional aspects of social isolation and frailty are essential to identifying those individuals in need of intervention. This suggests a practice and intervention focus on the deleterious effects on, and of, a lack of wider friendships and social organisations and frailty deficits, and the use of frailty deficits and a pre-disability clinical syndrome measures as markers for the risk of social isolation from social networks. Therefore, interventions targeting social isolation and frailty that consider an individual’s social placement in terms of isolation from friends and social organisations (e.g. facilitating individuals attending community events) may lessen some of the disadvantages caused by frailty, and which cause frailty. Furthermore, interventions that target physical exercise with friends and social organisations may do well at reducing both social isolation from friends and social organisations, and the accumulation of frailty deficits (physical inactivity and loss of muscle mass).

We suggest three limitations to our findings. Firstly, the conceptualisation of social isolation as comprising three dimensions from this existing database is limited to one- to two-item assessments of these factors. There is an opportunity now to explore ways of extending the measurement of these three dimensions so as to fully describe an individual’s level of social isolation across their family and social relationships, particularly in terms of whether that isolation is obligatory or voluntary. Secondly, the extent to which the findings are specific to this dataset and culture is unknown. Therefore, whether social isolation can be defined as three separate dimensions of social from “nuclear family”, “other immediate family”, and “a wider social network”, and the extent to which social isolation from a wider social network is related to frailty deficits needs to be examined in other cultural contexts. Thirdly, the current study follows previous studies (e.g. Gale et al. (2018)) by exploring variations in both social isolation and frailty at one data wave point, and exploring the relative influence on social isolation and frailty at a later data wave point. It may be useful to start the analysis at a time point at which a population is displaying an absence of social isolation and frailty and examine the extent to which the emergence of either social isolation or frailty is associated with the emergence of the other.

In summary, this study shows the importance of considering social isolation as multifactorial, reflecting dimensions of biological and social kinship. The findings also suggest that specificity, in focussing on social isolation from friends and social organisations, is useful for understanding the relationships between social isolation and frailty deficits longitudinally, and the Fried phenotype, as a critical threshold for the physiologic reserve and the beginning of the process of social isolation from wider social networks. This deeper understanding can be used to develop complex interventions that might impact social isolation and reduce frailty deficits in the longer term.

## Authors’ Note

### Open Data

Users registered with the Economic and Social Data Service (ESDS) have access to the English Longitudinal Study of Ageing datasets via www.esds.ac.uk.

#### Preregistration

The authors did not preregister the research with an analysis plan in an independent, institutional registry.
